# Synthesis of Nanoceria with Varied Ratios of Ce^3+^/Ce^4+^ Utilizing Soluble Borate Glass

**DOI:** 10.3390/nano12142363

**Published:** 2022-07-10

**Authors:** Kisa S. Ranasinghe, Rajnish Singh, Denis Leshchev, Angel Vasquez, Eli Stavitski, Ian Foster

**Affiliations:** 1 Department of Physics, Kennesaw State University, Marietta, GA 30060, USA; ifoster9@students.kennesaw.edu; 2 Department of Chemistry and Biochemistry, Kennesaw State University, Kennesaw, GA 30144, USA; rsingh@kennesaw.edu (R.S.); avasqu22@students.kennesaw.edu (A.V.); 3 Brookhaven National Laboratory NSLSII, Upton, NY 11973, USA; dleshchev@bnl.gov (D.L.); istavitski@bnl.gov (E.S.)

**Keywords:** glass, cerium(III)oxide, cerium(IV)oxide, nanoceria, soluble, borate glass, Ce^3+^/Ce^4+^

## Abstract

Mixed-valence cerium oxide nanoparticles (nanoceria) have been investigated with pronounced interest due to a wide range of biomedical and industrial applications that arises from its remarkable redox catalytic properties. However, there is no understanding of how to control the formation of these two types of nanoceria to obtain Ce^3+^/Ce^4+^ ratios required in various applications. In this work, using a soluble borate glass, nanoceria with specific ratios of Ce^3+^/Ce^4+^ are created and extracted via controlled glass-melting parameters. Glass embedded with nanoceria as well as nanoceria extracted from the glass were studied via XANES and fitted with the Multivariate Curve Resolution (MCR) technique to calculate the ratio of Ce^3+^/Ce^4+^. Results show that mixed-valence nanoceria with specific ratios are hermetically sealed within the glass for long durations. When the glass dissolves, the mixed-valence nanoceria are released, and the extracted nanoceria have unchanged Ce^3+^/Ce^4+^ ratios. Furthermore, TEM investigation on released nanoceria show that the nanoceria consist of several different structures. Although nanocrystal structures of Ce_7_O_12_, Ce_11_O_20_, and Ce_2_O_3_ contribute to the reduced state, a new quasi-stable phase of CeO1.66 has been observed as well.

## 1. Introduction

In recent years, increased interest has been given to nanoceria due to its remarkable catalytic and electronic properties acquired by alteration of the oxidation states; tetravalent Ce^4+^ (CeO_2_) and less stable trivalent Ce^3+^ (Ce_2_O_3_) through a reversible ceric–cerous redox equilibrium reaction [1]. A redox reaction between CeO_2_ and CeO_2−x_ through loss of oxygen and/or its electrons provides the cerium with the ability to reconfigure its electronic structure to adapt to the given environment. These oxygen vacancies in the lattice structure due to the reversible redox reaction make ceria a key ingredient for catalytic reactions, opening tremendous applications in industrial and medical fields. Nanoceria is applied in three-way catalytic converters, [2] solid oxide fuel cells (SOFC) [3], polymer electrolyte membrane (PEM) fuel cells [4], low-temperature ceramic fuel cells (CFC) [5], and nanoceria are used to create high-performing propellants [6]. There are several ongoing studies that demonstrate that controlling the Ce^3+^/Ce^4+^ ratios improve the properties of scintillators [7,8,9] and photodarkening in cerium-doped optical fibers [10]. In addition, ceria-doped glass is receiving significant attention due to their potential applications in non-linear materials for photonic devices [8,11,12]. The redox cycling between the two oxidation states plays a critical role in the use of nanoceria as scavengers of oxidizing radicals and molecules [7], alleviating pathologies associated with oxidative stress in biological systems [13]. Cerium-doped bioglass has antioxidant and anti-inflammatory properties and is useful in bone regeneration [14,15,16,17].

The importance of mixed-valence nanoceria is well characterized in biological systems. Hydrogen peroxide and superoxide anions are harmful byproducts of oxidative stress, and enzymes such as catalase and superoxide dismutase can quench these molecules, thus protecting the cell from oxidative stress damage [18,19]. Nanoceria with a predominance of Ce^4+^ mimics catalase while nanoceria with greater amounts of Ce^3+^ mimic superoxide dismutases [18,19]. Although these investigations underscore the importance of Ce^3+^/Ce^4+^ ratios, no studies have addressed controlling the Ce^3+^/Ce^4+^ ratio during synthesis, especially through glass formation with multivalent nanoceria embedded. In our previous work, we have shown that nanoceria of Ce^3+^ and Ce^4+^ valences are created when the glass is doped with CeO_2_ and can coexist within a soluble borate glass. The amounts of the two types of nanoceria created are controlled by glass-melting parameters, and when the glass dissolves in water, mixed-valence nanoceria of 2–5 nm are released into the aqueous media [20].

In the present study, we quantify the ratios sealed within the glass as a function of melting parameters and determine if the ratio of Ce^3+^/Ce^4+^ embedded within the glass remains the same when the nanoceria is extracted from the soluble glass. The transition of Ce^3+^ to Ce^4+^ is dynamic; however, when nanoceria of fixed ratios are created and embedded within a soluble borate glass we find that the Ce^3+^/Ce^4+^ ratio remains unchanged when the glass dissolves and nanoceria are released into the aqueous environment. The present study provides a novel mechanism to create mixed-valence nanoceria within a soluble glass and to extract mixed-valence nanoceria with the same Ce^3+^/Ce^4+^ ratio that was initially sealed within the glass.

## 2. Materials and Methods

### 2.1. Synthesis of the Glass Embedded Nanoceria

Sodium carbonate and diboron trioxide obtained from Alfa Aesar, MA, USA with 99.99% purity was mixed with powdered CeO_2_ of 99.9% purity to create a series of Na_2_O·2B_2_O_3_·*x*CeO_2_ glasses with x = 0.01–0.6 mol as shown in Table 1. The glass compositions containing 0.05 mol of CeO_2_ were then melted at different temperatures and times with different raw materials such as sodium tetraborate deca-hydrate to observe changes in Ce^3+^/Ce^4+^ ratios due to melting parameters. Each of these compositions were melted in a platinum crucible using a high-temperature furnace in air atmosphere and quenched between two steel plates to achieve a fast-cooling rate to prevent crystallization. Each sample was then processed to a fine powder using a Retsch Mixer Mill MM 500-Nanomill and stored in a desiccator cabinet. 

### 2.2. Extraction of Nanoceria

3.0 g of powdered glass samples were treated with 50 mL of DI water for different lengths of time. After the addition of water, samples were vigorously mixed and incubated in a shaker at 37 °C to completely dissolve the glassy matrix. The nanoceria from the glass was extracted by (a) allowing nanoceria to settle under gravity, (b) centrifugation at 1500 rcf (low speed) for 5 min or centrifugation at 14,000 rcf (high speed) for 30 min. In all instances, the supernatant is removed and the extracted nanoceria precipitate is dried overnight at 70 °C.

### 2.3. Characterization of the Glass and the Nanoceria

X-ray Absorption Near-Edge Spectroscopy (XANES) is used to determine the changes in the electronic structure of cerium within the glass as well as extracted nanoceria. XANES spectra were obtained at the Ce L_3_ edge (5.7 keV) in fluorescence mode for all the glass samples using the inner Shell Spectroscopy (ISS) beamline at the National Synchrotron Light Source NSLS II at Brookhaven National Lab. Ratios of Ce^3+^/Ce^4+^ within the glass as well as within the extracted nanoceria were resolved using XANES. To calculate the ratio of Ce^3+^/Ce^4+^, all XANES data were analyzed with Multivariate Curve Resolution (MCR) technique using PyMCR [21] implementation. The details of the analysis are summarized in the supporting information. Briefly, we first investigated the sensitivity of the fitting results to the reference spectra for Ce^3+/^Ce^4+^ species, as well as established a necessary set of constraints that yields physically meaningful results of the MCR fitting (Supporting information, Figure S1). Upon verifying that the procedure resulted in satisfactory fitting of the data (Supporting information, Figure S2), we have performed several runs of MCR fitting using different sets of reference spectra as starting solutions to recover the Ce^3+^/Ce^4+^ fractions in each sample (Supporting information, Figure S3). The reported Ce^3+/^Ce^4+^ fractions and their uncertainties were estimated based on averages and standard deviations of the MCR fitting runs. XANES is the most powerful technique to identify Ce^3+^/Ce^4+^ due to its ability to resolve differences at the absorption edge for different valence states of cerium ions. Other techniques such as Raman and UV-Vis spectroscopy are unable to provide accurate quantitative values for Ce^3+^/Ce^4+^ ratios as XANES does. Furthermore, the nanocrystalline structure was analyzed via a high-resolution FEI Tecnai G2 F30 Transmission Electron Microscope (TEM), Atlanta GA USA. TEM samples were prepared by resuspending the extracted nanoceria in DI water and the dispersed nanoparticles were placed on the TEM copper grid followed by overnight drying. The nanocrystalline structure was analyzed via Open-Source Image-J software. 

## 3. Results and Discussion

Our results show that when the glass composition is doped with cerium (IV) oxide, during high-temperature melting, ceria distributes mainly into two oxidation states, tetravalent Ce^4+^, and the less stable trivalent Ce^3+^. This formation of multivalent ceria nanoparticles (nanoceria) is governed by a cerox–ceric reaction, CeO_2_ ↔ CeO_2−__δ_ + $\frac{\delta }{2}$ O_2_ within the soluble borate glass. Depending on the oxygen availability in the molten state, the concentration of Ce^4+^ and Ce^3+^ changes. When the glass is formed, the tetravalent Ce^4+^ and less stable trivalent Ce^3+^ nanoceria, with specific ratios, are embedded and sealed within the glass indefinitely.

### 3.1. Ce^3+^/Ce^4+^ Ratio of the Nanoceria Embedded within the Glass

**Figure 1:**
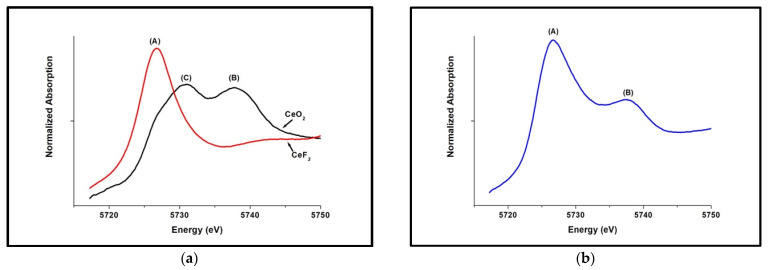
XANES spectrum for (**a**) standards of Ce(IV) and Ce(III) compounds, (**b**) Na_2_O·2B_2_O_3_ glass melted with 0.05 mols of CeO_2_.

**Figure 2:**
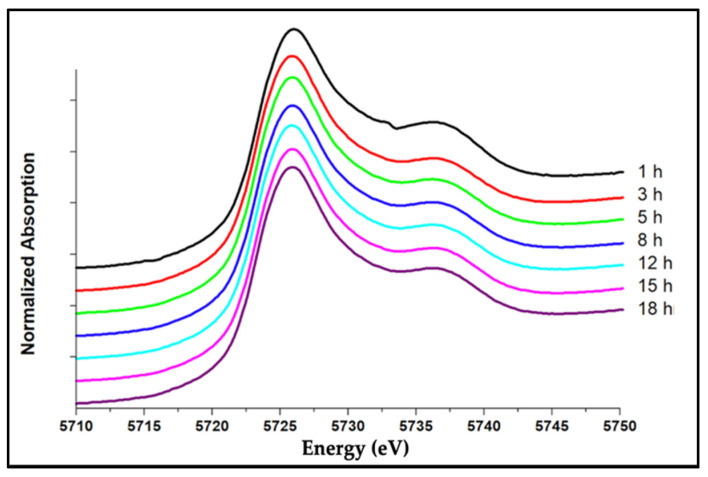
XANES spectrum for S5NBCe glass melted for different hours at 1100 °C.

To determine the cerium valence states in each of the melted glasses, in situ valence states of Ce^4+^ and Ce^3+^ were determined using XANES at the Ce L3 edge, which involves a 2p → 5d transition located around 5.7 keV. To identify the existence of Ce^4+^ and Ce^3+^ valences within the glass, cerium(III)fluoride (CeF_3_) and cerium(IV)oxide (CeO_2_) were used as standards. As shown in Figure 1a, the standard CeF_3_ shows a characteristic Ce^3+^ peak (A) at −5726 eV, and CeO_2_ shows two peaks around 5730 eV and 5737 eV. The peak B at 5737 eV is due to the excitation of an electron from 2p to 5d shell associated with the configuration 2p4f^0^5d^1^ whereas peak (C), at 5730 eV is due to the transition of an electron from the 2p orbital of adjacent O to the 4f shell of Ce to produce a final state of 2p4f^1^5d^1^ which is indicative of Ce-O bonding in CeO_2_ while the transition to 2p4f^1^5d^1^ is forbidden in cerium (III) oxide (Ce_2_O_3_) [17]. As shown in Figure 1b, when Na_2_O·2B_2_O_3_ glass composition is doped with 0.05 mols of CeO_2_, the Ce^4+^ ↔ Ce^3+^ reaction occurs to convert Ce^4+^ to Ce^3^ during the glass-melting process. As expected, the forbidden peak (C), at 5730 eV is notably missing within the glass. The two peaks were fitted with the MCR technique to calculate the ratio of Ce^3+^/Ce^4+^. Table 2 shows the Ce^3+^/Ce^4+^ ratios calculated by fitting XANES data using MCR for glass compositions with increased doping amounts of CeO_2_. The S4NBCe glass with 0.04 mol of CeO_2_ has the smallest Ce^3+^/Ce^4+^ ratio of 0.72, while S1NBCe glass with 0.01 mol of CeO_2_ melted at the same temperature and same time had the highest Ce^3+^/Ce^4+^ ratio of 2.41. Although we see shifts in Ce^3+^/Ce^4+^ ratios with different doping concentrations of initial CeO_2_, no specific trend that correlates Ce^3+^/Ce^4+^ratios to the doping content of CeO_2_ is observed. The Ce^3+^ concentrations in glass compositions doped with the same amount of CeO_2_ and melted at the same temperatures but for different times showed no significant change in the Ce^3+^/Ce^4+^ ratio, as shown in Figure 2. As seen in Table 3, when S5NBCe glass is melted with sodium tetraborate decahydrate at 1100 °C for from 1 h to 18 h, the Ce^3+^ concentrations remained near constant with a slight gain in the Ce^3+^ concentration from the first 1 h to 3 h of melting. When the same glass melted at temperatures 1200 °C and 1300 °C, the Ce^3+^ concentration is maintained at approximately 0.79 ± 0.09 and 0.87 ± 0.09, respectively. These results indicate that the equilibrium reaction that converts Ce^4+^ ⇾ Ce^3+^ occurs during the first few hours of melting. With no change in the furnace conditions, the longer hours of melting did not contribute to further catalytic conversion of ceria. Our results are comparable to the results obtained for an alkali borosilicate glass where Ce^3+^ concentration has remained at (83 ± 3)% when melted for 4 and 16 h [22]. On the other hand, as shown in Table 3, when the S5NBCe glass is melted at different melting temperatures for an hour, significant differences in Ce^3+^ concentrations are observed causing the ratio Ce^3+^/Ce^4+^ to change significantly. A similar trend was seen within S1NBCe glass showing an almost complete reduction to Ce^3+^. The S1NBCe, glass melted at 1200 °C for 1 h, and 93% of Ce^4+^ was reduced to Ce^3+^ giving a 13.13 ratio of Ce^3+^/Ce^4+^ while the same glass was melted at 1100 °C, only a 70% reduced to Ce^3+^ as shown in Table 4. However, when S1NBCe was melted at 1000° for 1 h, Ce^3+^/Ce^4+^ ratio was 8.82 with 90% of Ce^3+^ ions created by Ce^4+^ ⇾ Ce^3+^ transition during this melt. We believe when ceria undergoes thermal decomposition at high temperatures, there is a rapid decrease in Ce^4+^ with the formation of oxygen vacancies and a simultaneous formation of CO_2_ from sodium carbonate which can act as an oxidizer. Due to these competing processes, ceria could be partially reduced at 1100 °C while almost fully reduced at 1000 °C and 1200 °C. Schelter et al. [23] have discussed that ceria could be in multiconfigurational states, with some valences act as intermediate states that are trapped between Ce^3+^ and Ce^4+^ valent states. Our TEM data of the extracted nanoceria (see Section 3.3) strongly agree with this statement as we have observed the transformation of rhombohedral Ce_7_O_12_ to triclinic Ce_11_O_20_ at small temperature differences. We strongly believe that the redox changes between 1000 °C to 1100 °C and 1200 °C are a result of these intermediate states.

Furthermore, to study the effect of the furnace atmosphere and the raw materials on the formation of nanoceria with Ce^3+^/Ce^4+^ ratios, glass was melted at different air flow as shown in Table 4 while keeping the melting temperature at 1100 °C and 1 h of melting time constant. Results show that while melting in argon gas and dry air did not contribute to a significant change in Ce^3+^, but when melted with nitrogen gas atmosphere, 84% of the Ce^4+^ transferred to Ce^3+^. This could be because, while all three gases, nitrogen, argon, and dry air, were expected to be inert, nitrogen act as an inert gas at high temperatures. We used dry air to observe the difference between a normal air atmosphere within the furnace, but at high temperatures, due to the lack of water vapor, we did not observe any changes in Ce^3+^. Argon, being a noble gas, did not contribute any reduction or oxidization as expected. Furthermore, the glass was melted with different sources of cerium oxides as shown in Table 4, to understand the reduction process further. The glass melted with Ce(SO_4_)_2_ has almost the same amount Ce^3+^ while the glass doped with Ce(NO_3_)_3_ had a higher Ce^3+^. This result is as expected since the redox reaction, in this case, is Ce^3+^ → Ce^4+^ the abundance of oxygen makes it less likely to create Ce^4+^ nanocrystals. Additionally, we tested the shelf life of nanoceria-embedded glass to determine the effect of storage conditions on Ce^3+^/Ce^4+^ ratios within the nanoceria-embedded glass. We tested the S5NBCe glass that was melted and stored a year ago by XANES. Our results show (Table 4) that S5NBCe glass that was melted one year ago (labeled S5NBCe^*^) had roughly the same Ce^3+^ content, stating that the studied glass composition not only creates multivalent nanoceria but also maintains the ratio within the glass embedded indefinitely.

### 3.2. Ce^3+^/Ce^4+^ Ratio of the Extracted Nanoceria

**Figure 3:**
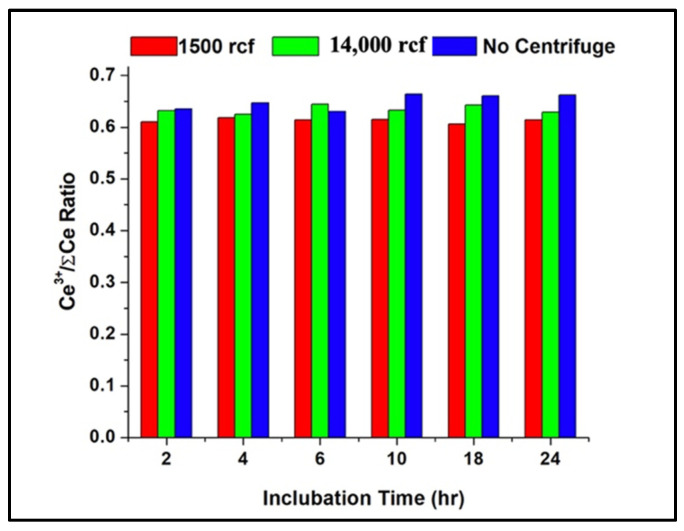
The Ce^3+^/Ce^4+^ of nanoceria extracted at 1500 rcf, 14,000 rcf, and no centrifuging from S5NBCe glass melted with sodium tetraborate decahydrate for 1 h at 1100 °C.

To determine if the released nanoceria from soluble glass maintains the same ratio that was created within the glass, we tested the extracted samples using XANES. The soluble borate glass S5NBCe was used, with the following differences. B_2_O_3_ and Na_2_CO_3_ were replaced with sodium tetraborate decahydrate. Melting was carried out at 1100 °C for 1 h. Prior to dissolution, the Ce^3+^ concentration for this glass was 0.64 ± 0.09 was determined by XANES, which is equivalent to Ce^3+^/Ce^4+^ ratio of 1.77 ± 0.51. To extract the nanoceria, the glass was incubated, with shaking, at 37 °C for various lengths of time from 2 h to 24 h. After incubation, nanoceria were collected with and without centrifugation. Two speeds of centrifugation were used, a low speed at 1500 rcf and a high speed at 14,000 rcf. Figure 3 shows the ratios of the concentration of Ce^3+^ analyzed by MCR data fitting of the XANES data for the extracted nanoceria. Within 2 h of incubation, the extracted nanoceria showed roughly the same amount of Ce^3+^ concentration with 0.61, 0.63, and 0.64 for 1500 rcf, 14,000 rcf, and zero rcf, respectively. Although both 1500 rcf and 14,000 rcf maintained the same Ce^3+^ amount within average, the nanoceria extracted using 1500 rcf had consistently a lower value averaging to a 0.61 ± 0.09 of Ce^3+^ amount with a lower Ce^3+^/Ce^4+^ ratio of 1.57 ± 0.48. When the nanoceria were extracted by just dissolving in DI water without centrifuging, the extracted sample showed a slight increase of Ce^3+^ amount with 0.66 ± 0.09 giving a Ce^3+^/Ce^4+^ ratio of 1.96 ± 0.44. The overall change in Ce^3+^ content in the extracted nanoceria is insignificant for the incubated times and but the speed of centrifugation has an effect. When the samples were incubated with no centrifugation, samples showed an increase in the Ce^3+^ content with longer incubation times. This could be because when collecting nanoceria without centrifugation, the cerous–ceric reaction is allowed to occur naturally in the presence of the glassy substrate where the, without any centrifugation, ceria may tend to reduce further increasing the Ce^3+^ concentration to form when compared to nanoceria collected by centrifugation. Collectively though, these results indicate that ratios of mixed-valence nanoceria extracted from this glass composition using different speeds of centrifugation are very stable, and even after 24 h of incubation in an aqueous media, the ratios remain the same.

### 3.3. TEM Analysis of Extracted Nanoceria

**Figure 4:**
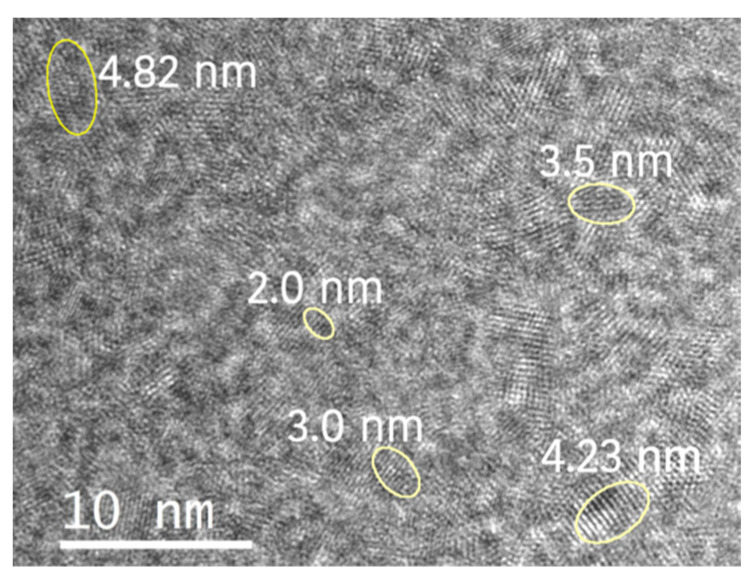
TEM image of nanoceria extracted from S5NBCe4 glass by dissolving in DI water for two hours. The marked crystals and their width is shown in the figure.

**Figure 5:**
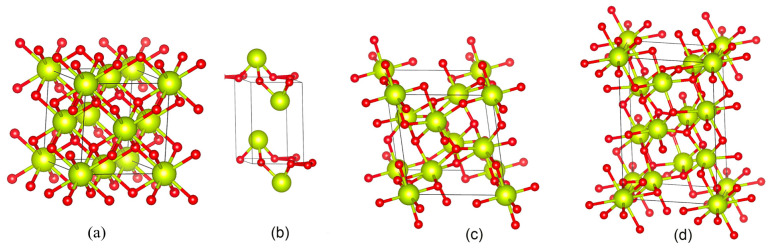
Schematic diagram of (**a**) cubic fluorite structure of CeO_2_, (**b**) hexagonal Ce_2_O_3_ structure, (**c**) rhombohedral Ce_7_O_12_, and (**d**) cubic body-centered structure of Ce_11_O_20_ larger cerium atoms are shown in green, while the smaller oxygen atoms are shown in red.

TEM results show that when powdered S5NBCe glass is dissolved in DI water for 2 h, the released nanoparticles are 2–5 nm in size, as shown in Figure 3. The oval shapes, seen in Figure 4, are drawn to highlight the nanoceria sizes. Even though the majority of the nanoceria are in the form of tetravalent Ce^4+^ (CeO_2_) and trivalent Ce^3+^ (Ce_2_O_3_); TEM data shows evidence of crystalline phases of Ce_7_O_12_ and Ce_11_O_20_. The schematic diagram of the unit cells of these identified structures are shown in Figure 5, which are plotted using Vesta software and the Crystallography Open database. The Ce^3+^ structures are observed in the form of Ce_2_O_3_, Ce_7_O_12_, and Ce_11_O_20_ as shown in Figure 5b–d, while the CeO_2_ phase is observed in the cubic fluorite structure as shown in Figure 5a [24,25,26].

**Figure 6:**
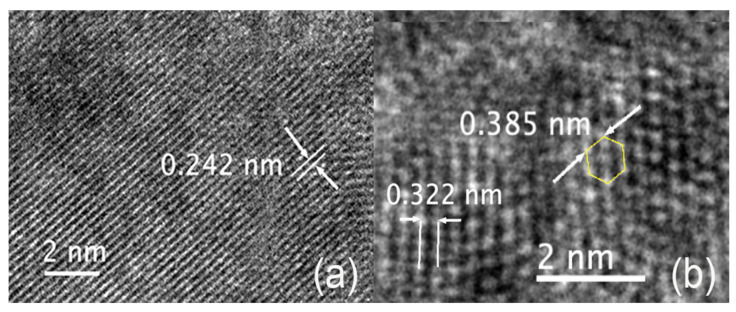
TEM image of the nanoceria obtained from the dissolution of S5NBCe glass with (**a**) the (100) plane of cubic CeO_2,_ (**b**) the (111) pane of cubic CeO_2_.

**Figure 7:**
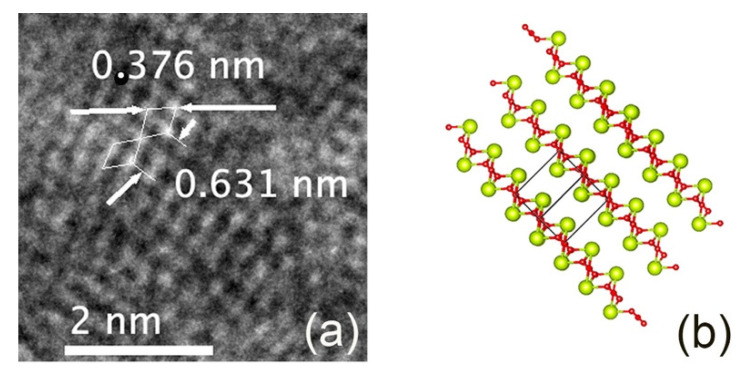
(**a**) TEM image of Ce_2_O_3_ nanoceria obtained from the dissolution of S5NBCe glass (**b**) the schematic diagram of A-type Ce_2_O_3_.

**Figure 8:**
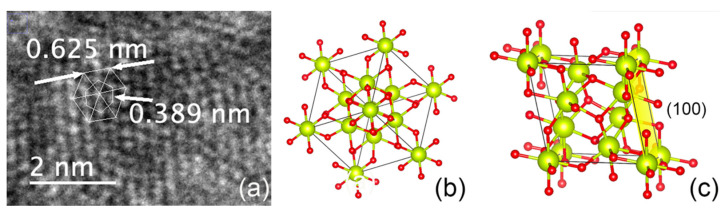
(**a**) TEM image from the dissolution of S5NBCe glass and the schematic diagram of Ce_7_O_12_ (**b**) cross-section of a (101) plane (**c**) rhombic unit cell.

The TEM images of the nanoceria particles obtained from the S5NBCe glass show nanoceria crystal structure with a lattice distance of 0.242 nm, as shown in Figure 6a, which is comparable to the theoretical value of the interatomic distance of Ce–O of the (100) plane of cubic CeO_2_ [27]. The lattice distance of 0.322 nm corresponds to the (111) plane of the cubic fluorite structure of CeO_2,_ as shown in Figure 6b. Our data on CeO_2_ structure are comparable to the nanoparticles synthesized by others [28,29,30,31], even though the nanoparticles created from our method are far smaller. The measured Ce–Ce of the interatomic distance of 0.385 nm matches to the distance between the face-centered cerium atom to the successive Ce atom of the cubic CeO_2_ of 0.3825 nm [27]. Out of the measured atomic distances in our nanoceria samples, a considerable amount of nanoceria has an atomic distance of 0.376 nm, which corresponds to the distance between Ce–Ce atoms on the (011) plane, as shown in Figure 7a. The measured distance between the two Ce atoms on the (100) plane is 6.31 Å which matches to the unit cell distance of the A-type Ce_2_O_3_. These measurements confirm the existence of the A-type Ce_2_O_3_ nanocrystals, as shown in the schematic diagram in Figure 7b. We have observed nanocrystals in the form of rhombic Ce_7_O_12_ and triclinic Ce_11_O_20_ in nanoceria extracted from the glass. Figure 8a shows a TEM image of the unit cell of Ce_7_O_12_ nanocrystal while that image was compared with the schematic diagram of Ce_7_O_12_ plotted according to the work done by Kümmerle et al. [24]. Image 7b shows the cross-section of a stackable (101) plane of the Ce_7_O_12_ unit cell. The atomic distance between the corner cerium atoms is 0.625 nm and comparable to the rhombic structure (Figure 8c), while the distance between the inner off-centered Ce atom is measured to be 0.389 nm.

**Figure 9:**
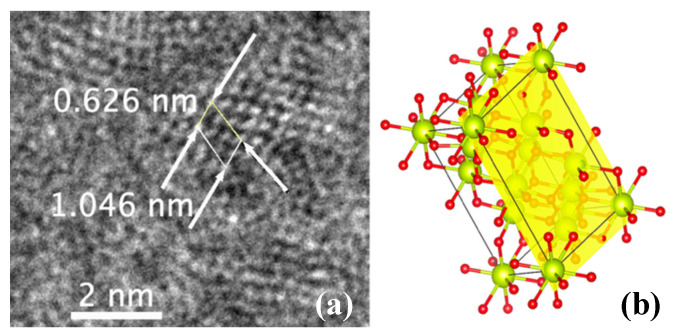
(**a**) TEM image of Ce_11_O_20_ nanoceria crystal (**b**) schematics diagram of the unit cell of Ce_11_O_20_.

Our measured distances also show us the existence of triclinic Ce_11_O_20_ nanocrystals. Figure 9a indicates a (100) plane of nanocrystal structure of Ce_11_O_20_ along with the schematic diagram of Ce_11_O_20_ schematic diagram of Ce_11_O_20_ with (110) plane indicated by the yellow.

A recent study conducted by Bekheed et al. discusses the coexistence of Ce_7_O_12_ and Ce_11_O_20_ on the reduced CeO_2_ when pure CeO_2_ powder is calcined at 1100 °C [32]. Their in situ high-temperature synchrotron XRD data indicated that CeO_2_ reduced to 62.4 wt.% rhombic-Ce_7_O_12_ and to 25.5 (0.6) wt.% triclinic Ce_11_O_20_. Bekheed et al. stated that the fluorite structure originally transformed into the bixbyite-type structure Ce_2_O_3+δ_ or CeO_2−δ_ around 955 °C, which stayed stable before transforming partially into rhombohedral Ce_7_O_12_ around 650 °C and to triclinic Ce_11_O_20_ at 520 °C while cooling down. They claimed that the Ce_7_O_12_ consists of Ce_0.58_^3+^Ce_0.42_^4+^O_1.71_ while the Ce_11_O_20_ phase consists of Ce_0.48_^3+^Ce_0.52_^4+^O_1.76_. Furthermore, Murgida et al. [33] argued that the crystal structures of Ce_7_O_12_, Ce_11_O_20_, and C-type Ce_2_O_3_ are reduced structures that can be considered to be sublattices of CeO_2_ fluorite.

**Figure 10:**
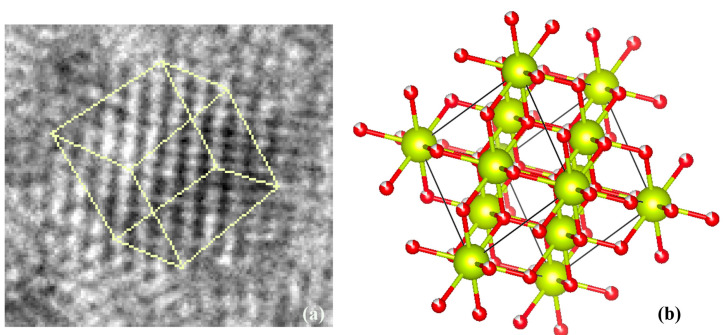
Schematics diagrams of (**a**) FCC structure of CeO_1.66_ and (**b**) schematics lattice structure of CeO_1.66_.

These results confirm our data as we believe the abundance of oxygen in the glass matrix reduces the CeO_2_ and further changes the phase as the melt cools down, giving rise to various Ce^3+^/Ce^4+^ ratios depending on the glass-melting parameters. We also observed another phase with a structure of CeO1.66 that has been newly identified [33,34] as a possible reduced phase. Some of the TEM images of nanoceria crystals analyzed show measured Ce-Ce lattice distances of 0.398 nm and 0.559 nm, indicating another FCC structure with Ce-O lattice distance of 0.246 nm, as shown in Figure 10a. These lattice parameters suggest the existence of nanoparticles in the form of CeO1.66 crystal, as shown in Figure 10b, schematics lattice structure plotted according to the theoretical data [24]. Murgida et al. [33] discussed a quasi-stable bulk Ce_3_O_5_ structure that is equivalent to the CeO1.66 structure that we have observed within the nanoceria extracted from the glass. These results indicate the formation of a mixed-valence state of nanoceria within the glass during melting and release; nanoceria acquired from the glass consists of multivalent nanoceria with different Ce^3+^/Ce^4+^.

Furthermore, it has been observed that the nanoparticles that are less than 5 nm undergo lattice expansion [35,36,37,38,39,40,41]. According to Tsunekawa et al., when nanoceria undergoes a redox reaction, the CeO_2_ is reduced to CeO_2−x_ state and has been associated with a lattice expansion as the smaller Ce^4+^ transforms to a larger ionic radius such as Ce^3+^ [42]. Furthermore, Diehm et al. discussed that the reason for a lattice expansion is due to the surface stress of the nanoparticle [43]. The data on lattice expansion on the CeO_2_ is quite contradictory, while the majority of the data shows a 0.1% to 0.5% lattice expansion [9], Tsunekawa et al. [42] and Wu et al. [44] showed a 2.5% to 3.5% lattice expansion. However, the data published by Hailstone et al. suggested a 7% lattice expansion on CeO_2,_ while the theoretical work done by Sayle et al. [45] discusses the possibility of a 10% reduction on the lattice parameter. The experimental study conducted by Wang et al. [46] on the CeO_2_ nanoparticles obtained from the hydrothermal process did not show any significant difference in the lattice parameters. We observed a 0.653% lattice expansion in the most stable (111) plane of the CeO_2_ crystal. As shown in Table 5, we have observed a considerable expansion in the primary axis of the unit cell of Ce_2_O_3_ nanoparticle while the distance between the two cerium atoms on the same plane is reduced by 0.262%. A similar expansion was observed in the distance between two consecutive cerium atoms on the same plane of Ce_11_O_20_ nanoparticles, but we have observed a 6.298% reduction in the primary axis of the unit cell of Ce_7_O_12_ nanoparticle from that of the theoretical value of the bulk. The distance between two consecutive cerium atoms on the same plane of Ce_7_O_12_ nanoparticle was reduced as well by 0.262%. We believe the reduction is due to the coexistence of both Ce^3+^ and Ce^4+^ phases due to oxygen vacancy. The literature only discussed the lattice expansion of ceria nanoparticles in the form of CeO_2_ nanocrystal to its reduced state, and our data are comparable to the majority of the published data on the lattice expansion [9]. There are no data on the literature on the lattice change in Ce_7_O_12_ and Ce_11_O_20_, nanoparticles for comparison.

## 4. Conclusions

We have successfully synthesized nanoceria with varied ratios of Ce^3+^/Ce^4+^ ratios using a soluble borate glass. Although the glass-melting temperature variations changed the Ce^3+^/Ce^4+^ ratios, the duration of melting did not contribute to any significant change. When a specific glass composition with identified Ce^3+^/Ce^4+^ ratio was dissolved in DI water, extracted nanoceria maintained the same ratio of Ce^3+^/Ce^4+^ as that of the initial glass. The incubation times in DI water had no significant change in the ratios, and neither did the speed of centrifugation. The extracted nanoceria exhibited multivalency and the three phases, Ce_2_O_3_, Ce_7_O_12,_ and Ce_11_O_20_, contributed to the Ce^3+^ content within the nanoceria. Furthermore, we have observed a Ce_3_O_5_ phase that has been identified as the quasi-stable state in the extracted nanoceria. Lastly, we have confirmed that the Ce^3+^/Ce^4+^ ratio of the nanoceria embedded in the glass remained unchanged even after a year of melting.

## 5. Patents

US and International Patent-065848.0030PCT1, Ranasinghe, K.S, Singh, R., Disclosure of the invention: Multivalent Cerium oxide Nanoparticles in Soluble Borate Glass Matrix for Targeted Release. (April 2020).

## Figures and Tables

**Table 1:** Composition/Identification for glasses Na_2_O·2B_2_O_3_ ^1^ doped with different amounts of CeO_2_.

Sample	CeO_2_ Concentration	Melting Temperature	Melting Time
S1NBCe	0.01 mol	1100 °C	1 h
S2NBCe	0.02 mol	1100 °C	1 h
S3NBCe	0.03 mol	1100 °C	1 h
S4NBCe	0.04 mol	1100 °C	1 h
S5NBCe	0.05 mol	1100 °C	1 h
S6NBCe	0.06 mol	1100 °C	1 h

^1^ All the samples were melted in air atmosphere with raw materials Na_2_CO_3_ and B_2_O_3_ to obtain Na_2_O·2B_2_O_3_ glass.

**Table 2:** The concentrations of Ce^3+^ and Ce^3+^/Ce^4+^ ratio within the processed glasses Na_2_O·2B_2_O_3_ ^1^ melted with different amounts of CeO_2_.

Sample	CeO_2_ mols	Ce^3+^/ΣCe (±0.09) ^2^	Ce^3+^/Ce^4+^
S1NBCe	0.01	0.71	2.41 ± 0.80
S2NBCe	0.02	0.61	1.55 ± 0.42
S3NBCe	0.03	0.61	1.55 ± 0.42
S4NBCe	0.04	0.42	0.72 ± 0.19
S5NBCe	0.05	0.58	1.38 ± 0.37
S6NBCe	0.06	0.63	1.72 ± 0.49

^1^ All the samples were melted in air atmosphere at 1100 °C for one hour and doped with Ce (IV) O_2_. ^2^ The XANES data were rounded to two decimals as the MCR data have ±0.09 error with a 95% confidence level.

**Table 3:** The concentrations of Ce^3+^ with different melting times at different temperatures for S5NBCe glass melted with sodium tetraborate decahydrate.

Melted Time (h)	Ce^3+^ /∑Ce (±0.09) (1100 °C)	Ce^3+^ /∑Ce (±0.09) (1200 °C)	Ce^3+^ /∑Ce (±0.09) (1300 °C)
1	0.62	0.77	0.87
3	0.70	0.79	0.87
5	0.69	0.80	0.87
8	0.68	0.80	0.88
12	0.69	0.78	-
15	0.668	0.80	0.88
18	0.69	-	-

**Table 4:** The concentrations of Ce^3+^ and Ce^3+^/Ce^4+^ ratio within the processed glasses Na_2_O·2B_2_O_3_ melted with different melting variables.

Sample	Cerium Source	Concentration (mol)	Temperature	Time	Furnace Atmosphere	Ce^3+^ /∑Ce (±0.09)
S1NBCe	Ce(IV)O_2_	0.01	1000 °C	1 h	air	0.90
S1NBCe	Ce(IV)O_2_	0.01	1100 °C	1 h	air	0.70
S1NBCe	Ce(IV)O_2_	0.01	1200 °C	1 h	air	0.93
S1NBCe	Ce(IV)O_2_	0.01	1100 °C	2 h	air	0.70
S1NBCe	Ce(IV)O_2_	0.01	1100 °C	3 h	air	0.70
S5NBCe	Ce(IV)O_2_	0.05	1100 °C	1 h	Dry air	0.64
S5NBCe	Ce(IV)O_2_	0.05	1100 °C	1 h	Ar	0.68
S5NBCe	Ce(IV)O_2_	0.05	1100 °C	1 h	N_2_	0.84
S5NBCe	Ce(NO_3_)_3_	0.05	1100 °C	1 h	air	0.77
S5NBCe	Ce(SO_4_)_2_	0.05	1100 °C	1 h	air	0.56
S5NBCe	Ce(IV)O_2_	0.05	1100 °C	1 h	air	0.57
S5NBCe *	Ce(IV)O_2_	0.05	1100 °C	1 h	air	0.58

^*^ S5NBCe ^*^ was melted and kept under a desiccator for one year.

**Table 5:** The Ce lattice distance of nanoceria extracted from S6NBCe4 glass compared with the theoretical values.

Nanocrystal	Ce-Ce Lattice Distance Measured (Å)	Ce-Ce Theoretical Lattice Distance (Å)	Lattice Expansion
CeO_2_	(3.85 ± 0.06)	3.825 [26]	+0.653%
Ce_2_O_3_	(3.76 ± 0.04) and (6.31 ± 0.07)	3.77 and 6.06 [25]	−0.398% and +4.175%
Ce_7_O_12_	(6.35 ± 0.08) and 3.80 ± 0.09)	6.78 and 3.812 [24]	−6.298% and −0.262%
Ce_11_O_20_	(3.73 ± 0.01)	3.717 [24]	+0.215%

## Data Availability

No data reported other than what presented.

## Supplementary Materials

The following supporting information can be downloaded at: https://www.mdpi.com/article/10.3390/nano12142363/s1, Figure S1: Spectra obtained from the MCR procedure using different combinations of reference spectra as starting solutions. (left) spectra obtained from the MCR fitting when both spectral components are allowed to vary freely. (right) spectra obtained from the MCR fitting when Ce^4+^ spectra are fixed; Figure S2: Comparison between the data and the MCR fit using CeO_2_ and CeF_3_ spectra as starting solutions. Similar fits were obtained for other starting solution sets; Figure S3: Comparison between the Ce^3+^/Ce^4+^ fractions for the sample series was obtained using different starting solutions. Dashed and solid lines correspond to fractions of Ce^3+^ and Ce^4+^, respectively
