# Supplementary material for: Synthesis of Nanoceria with Varied Ratios of Ce3+/Ce4+ Utilizing Soluble Borate Glass
Source: Nanomaterials (Basel). 2022 Jul 10;12(14):2363. doi: 10.3390/nano12142363 (PMC9323119; doi:10.3390/nano12142363)
Supplement: Supplementary file 1 [file nanomaterials-12-02363-s001.zip › nanomaterials-1746287-supplementary.pdf]

Extracting Ce3+/Ce4+ concentrations using Multivariate Curve Resolution of XANES data.

Alternating Least-Squares Multivariate Curve Resolution (ALS-MCR) implemented in pyMCR library was employed to analyze the compositions of the sample series [1]. Compared to traditional Linear Component Fitting (LCF), ALS-MCR fits not only the amplitudes of the species contributions, but also refines the spectral shapes, allowing extraction of more representative spectral profiles and concentration distributions from the data. The ALS-MCR fitting was initialized by supplying two spectra recorded on Ce^{3+} and Ce^{4+} reference compounds. In this work, we performed several runs of ALS-MCR fitting using different combinations of reference spectra of (CeO_2/CeF_3), (CeO_2/$CePO_4$), ($CeSO_4$/CeF_3), and ($CeSO_4$/$CePO_4$), which allowed us to evaluate the robustness of the fitting and uncertainty of species concentrations. Initially, we attempted to fit the data by using non-negativity constraints on component spectra and concentrations, as well as fixing the total amount of Ce^{3+}/Ce^{4+} to 1. As shown in Figure S1a, we observe that when $CeSO_4$ spectrum is used as a reference for Ce4+ the fit yields a Ce^{4+} species spectrum with an unphysical dip around 5723 eV. This dip is close to the Ce^{3+} species maximum at 5725 eV, indicating that spectral features of two species are strongly correlated in this region. In addition, when we use CeO_4 as a starting guess, the retrieved spectrum for the Ce^{4+} species very closely resembles that of the $CeSO_4$ spectrum. In case of all starting spectra pairs, the Ce^{3+} species spectrum appears to be qualitatively the same, although some variation in the intensity of the main peak at 5725 eV is observed. To avoid obtaining unphysical spectral features, as in case when $CeSO_4$ spectrum is used as a starting solution, we performed the second run of MCR optimization where we constrained the Ce^{4+} spectrum and allowed Ce^{3+} to vary, while maintaining the rest of the constraints as before. The spectra do not possess non-physical features indicating that that the obtained solutions are representative of the species present in the sample. The comparisons of typical fits with the data are shown in Figure S2 and demonstrate satisfactory fit quality; these comparisons are representative of the fits obtained using different starting spectra. We note that some of the minor discrepancies between the data and fit may be attributed to existence of additional species differing in their local environment, as evidenced by the TEM results presented in the main text. Figure S3 shows the fractions of species obtained from the fitting. We observe that different starting solutions yield different concentration values. These variations were used to evaluate the uncertainty in the retrieved concentrations. The fractions, reported in the main text, were computed as average of the MCR runs. The uncertainty of the retrieved fractions was obtained using the standard deviation

computed from different runs and is reported as 2σ, corresponding to the 95% confidence interval. We found that all samples exhibited approximately the same level of uncertainty of 0.09.

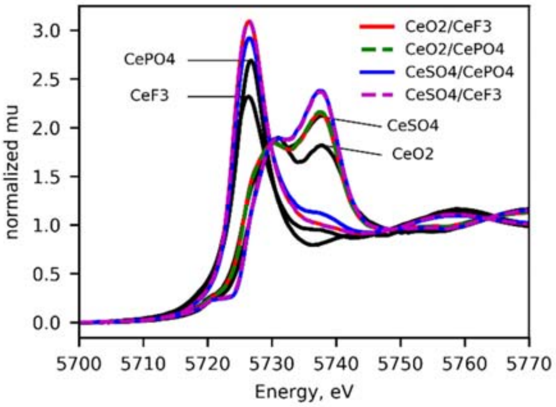

Figure S1. Spectra obtained from the MCR procedure using different combinations of reference spectra as starting solutions. (left) spectra obtained from the MCR fitting when both spectral components are allowed to vary freely. (right) spectra obtained from the MCR fitting when Ce^{4+} spectra are fixed.

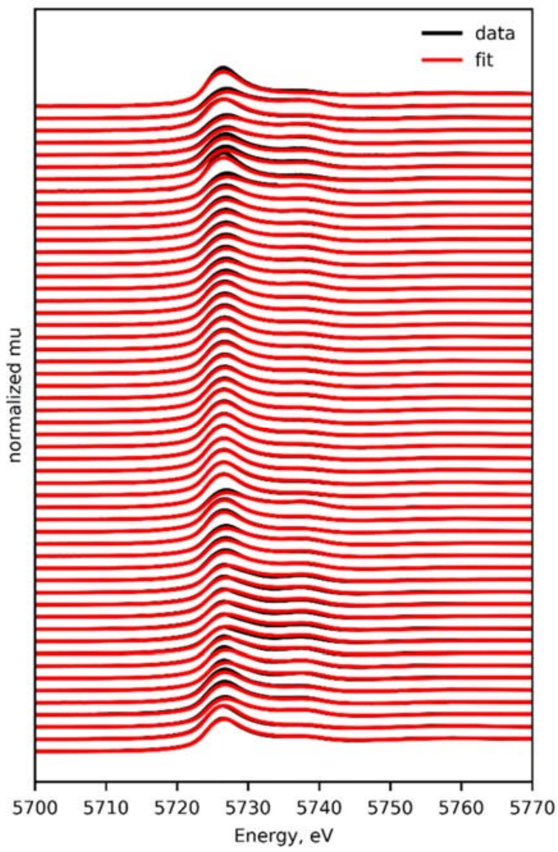

Figure S2. Comparison between the data and the MCR fit using CeO_2 and CeF_3 spectra as starting solutions. Similar fits were obtained for other starting solution sets.

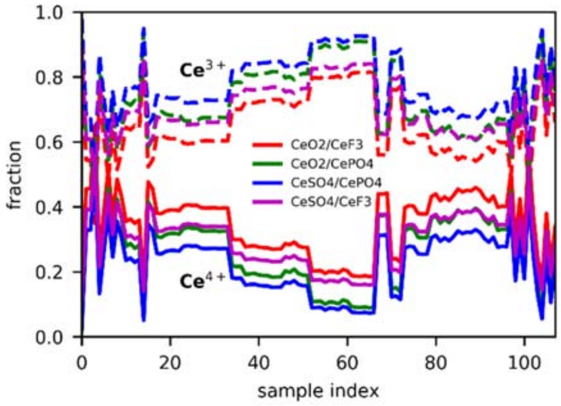

Figure S3. Comparison between the Ce^{3+}/Ce^{4+} fractions for the sample series was obtained using different starting solutions. Dashed and solid lines correspond to fractions of Ce^{3+} and Ce^{4+}, respectively.

References

[1] C. H. Camp, "PyMCR: A python library for multivariatecurve resolution analysis with alternating regression (MCR-AR)," *J. Res. Natl. Inst. Stand. Technol.*, vol. 124, no. 124018, pp. 1–10, 2019, doi: 10.6028/jres.124.018.
